# The Administration of Anæsthetics to Children

**Published:** 1908-08-01

**Authors:** 


### Anæsthetics.

### THE ADMINISTRATION OF ANÆSTHETICS TO CHILDREN.

In selecting an anæsthetic for a child it is necessary to take into account, no less than in the case of adults, such circumstances as the nature and probable duration of the operation, and the condition of the patient.

For short operations, such as opening an abscess, nitrous oxide is not always so satisfactory as in adults. A young or nervous child is apt, in spite of encouragement, to become alarmed at the outset and require forcible restraint. The narcosis is often accompanied by inconvenient rigidity or jactitation, and sometimes by involuntary evacuations. On recovery there is apt to be renewed screaming and struggling, which does not always indicate consciousness of pain, but is distressing, and interferes with the toilet of the wound or other necessary proceedings. Ethyl chloride gives a quieter anæsthesia, but it is open to the same objections as nitrous oxide, and is undoubtedly more dangerous: there is also more liability to sickness, restlessness, and headache afterwards. It is extensively used for the removal of tonsils and adenoids, partly because (in hospital practice) it allows a considerable saving of time, partly because it allows the patient to be in the sitting position, which is greatly preferred by some operators. In most cases, *provided the operator be expert and the anæsthetist have had special experience*, it answers extremely well, but if enough anæsthetic has not been given or if any hitch occurs (such as the escape of a tonsil from the guillotine) the child will probably come round and struggle before the operation is properly finished, and if the anæsthesia be a little too deep there is serious risk from blood accumulating in and above the larynx in consequence of the abolition of the "cough-reflex," or from muscular rigidity. The anæsthetist, besides keeping the gag in position, must push the tonsils inwards and upwards and hold the head steady, tilting it forwards as opportunity allows; if the breathing stops more than momentarily, or if cyanosis is evident by the colour of the blood and lips, the gag must be at once loosened, the head tilted forward, the pharynx cleared, and the usual measures taken, as far as may be necessary, to restore respiration.

The administration of ether is liable to be followed by bronchial catarrh in infants and young children, and it should not be given at any age when there is respiratory difficulty from obstruction or disease, nor when danger to the patient or inconvenience to the operator will be caused by vascular engorgement, secretion of mucus, or deep breathing. It is, therefore contraindicated, for example, in tracheotomy, operations on the brain, and usually in abdominal operations. It should be preceded by $N_2O$, $C_2H_5Cl$, or A.C.E. and free admission of air allowed as soon as induction is completed.

A.C.E. or C.E. is a pleasant and (when carefully given) a safe anæsthetic, and may be employed in most cases. It should be given gradually and steadily, no large quantity being poured on the sponge: the mask should be frequently removed, thus avoiding concentration of vapour, and the pupils and breathing constantly watched, an insidious failure of this function, with dilatation of the pupils, being the usual result of an overdose.

Chloroform may be given with convenience and safety in a large proportion of cases, but unremitting watchfulness is necessary during its administration, and its use for debilitated children is much more dangerous than is commonly supposed—particularly in operations such as circumcision, which may cause shock or laryngeal spasm. It is most important that plenty of air be allowed. The edges of the lint, if used, should never be closely applied to the face. Schimmelbusch's mask is preferable to Skinner's, as there is less risk of contact shutting off air, and it also provides more effectually against blistering. Induction should be gradual and steady, a few drops being given at first and the amount increased in definite proportion and at definite intervals until anæsthesia is established. When given in this way practical experience only can determine the amount and rate advisable in any individual case, and ensure the avoidance of timidity on the one hand or recklessness on the other.

This is not an occasion to discuss the merits and demerits of the more complicated percentage-inhalers, but mention may be made of a very simple one (Krohne and Sesemann's modification of Junker's), in which there is complete avoidance of respiratory effort. Chloroform is much economised by its use. If the child is amenable induction is very satisfactory: the vapour does not irritate the eyes (crying is often thus started in children when the usual lint is employed); but if there is rapid breathing or struggling it is likely to be difficult and exhausting, and it is better to use lint or a simple mask until the child is quiet.

The writer is unable to say whether the claims of the makers in regard to accurate percentages should be accepted; but even if they are correct the anæsthetist must trust to his own judgment and observation rather than to the apparatus: he must use the minimum strength required by the case, and he should frequently remove the mask from the face in order that a complete renewal of air may be effected.

Operations should, if possible, be arranged to take place about the time when the mid-day or evening meal would otherwise occur. Children are easily upset by an alteration in their usual habits, *i.e.*, changing the time of feeding to fit the operation, and they are not in such good condition as adults to take an anæsthetic after a night's fasting. Four or five hours' interval can thus be obtained. A shorter fast is advisable in cases of infants or weakly children. Care must be taken that nothing which takes long to digest, such as currant buns, is given at the last meal, and it is advisable to take the same precautions for $N_2O$ or ethyl chloride as for other anæsthetics.

The anæsthetist must take a general and, if necessary, a detailed survey of the patient. Attention should be especially directed to pallor due to disease or to fright, and to signs of rickets, indicating defective respiration and tendency to laryngeal spasm. The mouth must be examined for loose teeth—sometimes sweets are discovered! In mouth-breathers a prop or gag should be inserted, but not opened to the fullest extent. Care must be taken not to nip the lips or tongue, nor to push down the latter.

If lint or a simple mask be used the child should be told to shut the eyes as if going to sleep, and various means may be employed to obtain quietude and steady breathing. Nursery rhymes may be repeated to little children, and older ones may be told to count slowly. If struggling and crying occur one should go on steadily, but without pushing the anæsthetic, especial care being taken not to allow a sudden over-dose when there is holding of the breath followed by deep inspiration. The head should always be turned on one side, and after induction has been accomplished it is generally advisable to push forward the lower jaw by pressure at one or both angles, which carries forward the tongue and frees the glottis. Forceps should never be used to hold out the tongue longer than momentarily. If necessary to continue this should be done by the fingers or by putting in a ligature. During induction, disturbance by removal of bandages, clattering of instruments, etc., should not be allowed, and the operation should not be begun until the anæsthetist considers the patient ready. Under too light anæsthesia an incision or even the passage of a catheter may have a serious effect.

The corneal reflex in children is very unreliable; other signs must be depended upon as regards depth of anæsthesia—the character of the breathing, flaccidity of limbs, absence of response to pinching or to rubbing the lower part of the chest. The degree of anæsthesia needs careful regulating according to the operation and the stage at which it has arrived.

If the prone or latero-prone position be necessary, the anæsthetist must carefully watch its effect on the respiration, especially when, as in infants and rickety children, there is tumidity of the abdomen and weakness of the thoracic walls. In cases of empyema the child should be held with the affected side projecting over the edge of the table, or else turned on this side in the latero-prone position. Large empyemata should be aspirated beforehand.

Attention has in the last few years been drawn to the "status lymphaticus" as one in which the administration of an anæsthetic is peculiarly dangerous. The reports usually indicate, however, that other factors such as respiratory obstruction, premature commencement of the operation under imperfect anæsthesia, etc., had at least a share in the fatalities. If this condition be suspected, even more than usual care must be taken, and, if possible, ether given rather than chloroform or a mixture.

Much has also been written on the subject of so-called "delayed chloroform poisoning." The essential cause of this would appear to be some antecedent condition not yet fully understood. If this be indicated beforehand by such signs as vomiting, acetonuria, or enlarged liver, no operation requiring a general anæsthetic should be performed unless urgent. It must always be remembered that continuous vomiting after an operation may be due not only to the above, but to a variety of causes quite apart from the anæsthetic; for instance, insufficient preparation, direct or reflex disturbance of internal organs, etc., etc.; putting these aside, very much depends on special experience of the administrator and on the care with which it is applied.

